# Macrophages: plastic participants in the diagnosis and treatment of head and neck squamous cell carcinoma

**DOI:** 10.3389/fimmu.2024.1337129

**Published:** 2024-04-08

**Authors:** Chen Lin, Yidian Chu, Ye Zheng, Shanshan Gu, Yanghao Hu, Jiali He, Zhisen Shen

**Affiliations:** ^1^ The Affiliated Lihuili Hospital, Ningbo University, Ningbo, China; ^2^ Health Science Center, Ningbo University, Ningbo, China

**Keywords:** head and neck squamous cell carcinoma, macrophages, tumor microenvironment, immunotherapies, engineering macrophages, CAR-M cell therapy

## Abstract

Head and neck squamous cell carcinoma (HNSCC) rank among the most prevalent types of head and neck cancer globally. Unfortunately, a significant number of patients receive their diagnoses at advanced stages, limiting the effectiveness of available treatments. The tumor microenvironment (TME) is a pivotal player in HNSCC development, with macrophages holding a central role. Macrophages demonstrate diverse functions within the TME, both inhibiting and facilitating cancer progression. M1 macrophages are characterized by their phagocytic and immune activities, while M2 macrophages tend to promote inflammation and immunosuppression. Striking a balance between these different polarization states is essential for maintaining overall health, yet in the context of tumors, M2 macrophages typically prevail. Recent efforts have been directed at controlling the polarization states of macrophages, paving the way for novel approaches to cancer treatment. Various drugs and immunotherapies, including innovative treatments based on macrophages like engineering macrophages and CAR-M cell therapy, have been developed. This article provides an overview of the roles played by macrophages in HNSCC, explores potential therapeutic targets and strategies, and presents fresh perspectives on the future of HNSCC treatment.

## 1 Introduction

Head and neck squamous cell carcinoma (HNSCC) is the most prevalent form of head and neck cancer and ranks as the seventh most common cancer worldwide (1). Regrettably, most instances are detected at advanced stages, often involving locally advanced (LA) conditions or distant metastasis (DM). Despite the availability of various treatment options, such as surgery, radiation therapy (RT), chemotherapy (CT), and immunotherapy (IT), a significant portion (40-60%) of LA tumors eventually experience relapse or local progression. Palliative CT for metastatic and recurrent (R/M) HNSCC tumors also presents a grim prognosis (2). The tumor microenvironment (TME) refers to the immediate surroundings of HNSCC tumors during their growth or mutation, exhibiting complexity. On one hand, alterations such as cytokine production and extracellular matrix changes occur within the TME, alongside immune surveillance which identifies and attacks tumor cells, thereby inhibiting tumor growth. On the other hand, tumor cells can interact with surrounding tissues to modify nutrient supply, generate cytokines, and suppress immune responses within the TME, thus promoting their own survival and development (3, 4).

**Figure 1:**
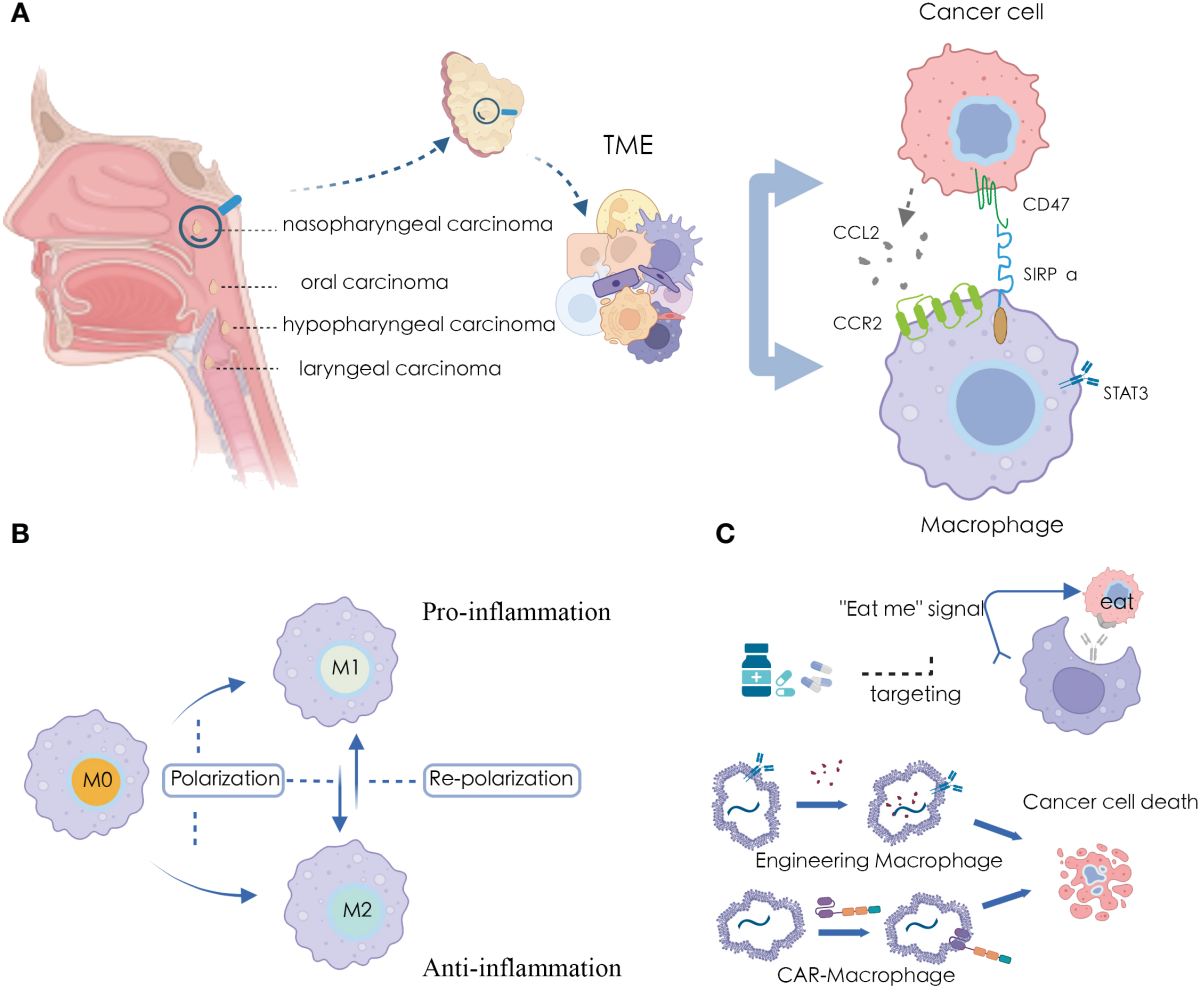
Macrophages and applications in HNSCC microenvironment **(A)** Significant crosstalk between macrophages and tumor cells in HNSCC TME; **(B)** The plasticity of macrophage polarization states; **(C)** Targeting and harnessing macrophages in disease treatment and immunotherapy.

Most patients diagnosed as HNSCC often present with locally advanced disease, requiring multimodal treatments, including immunotherapy (5, 6). Immunotherapy involves the specific recognition and targeting of cancer cells by immune cells within the TME. The TME contains various immune cells such as macrophages, effector T cells, natural killer cells, and dendritic cells (7). Among them, macrophages constitute the largest and most critical group of innate immune cells in the TME (8) (**Figure 1A**). Macrophages originate from bone marrow hematopoietic stem cells and embryonic yolk sac tissue (9, 10). Under the influence of different microenvironmental stimuli, macrophages exhibit heterogeneity and plasticity, allowing them to adapt their characteristics in highly specialized ways to perceive and respond to their environment. While the specific phenotypes are hard to categorize, they can be simplified into two extremes with entirely different molecular phenotypes and functional characteristics: IFN-γ/lipopolysaccharide (LPS)-induced M1 macrophages and IL-4/IL-10/IL-13-induced M2 macrophages (7).

Furthermore, tumor-associated macrophages (TAMs) refer to a type of macrophages that appear in the TME that exhibit characteristics of both M1 and M2 macrophages under different signals and stimuli (11, 12). Among these, in the TME, typical M1 activators include LPS, IL-1β, and IFN-γ, which activate macrophages to produce inflammatory factors. Conversely, M2 polarization factors such as IL-4, IL-13, IL-10, and TGF-β promote macrophage polarization towards the M2 type, exhibiting characteristics of anti-inflammatory and immune suppression (13). In HNSCC, TAMs typically display M2 macrophage features, contributing to the establishment of an immune-suppressive TME, thereby promoting tumor escape and growth (12, 14). The balance between macrophage M1 and M2 subtypes is crucial for maintaining a stable state of health in the human body. When this balance is disrupted, it can lead to disease states (15). Metabolic adaptation supports the heterogeneity of tumor-associated macrophage activities and functions, maintaining their polarization in specific environments (16, 17). In particular, in terms of energy supply, M1 TAMs primarily rely on glycolysis. The two interruptions in their TCA cycle lead to the accumulation of itaconate and succinate, resulting in the stabilization of HIF1α. This further activates the transcription of glycolytic genes, thereby maintaining the glycolytic metabolism of M1 cells (18). Conversely, M2 cells are more dependent on oxidative phosphorylation, with their TCA cycle intact and providing substrates for the electron transport chain complexes (18). Additionally, both subtypes have specific metabolic pathways that regulate lipid and amino acid metabolism, influencing their responses (19, 20).

Due to the high plasticity of macrophages, they play a significant role in various pathological processes, further exploring macrophages is considered as an alternative approach for cancer therapy (21). Recent studies have indicated that promoting M1 macrophage polarization and inhibiting M2 macrophage polarization can exert anti-tumor effects, while promoting M2 macrophage polarization and inhibiting M1 macrophage polarization can have anti-inflammatory activity (22) (**Figure 1B**). Several drugs have been discovered that can modulate the polarization state of macrophages for disease treatment (23, 24), and many immunotherapeutic approaches have been developed using macrophages as carriers or tools (25, 26) (**Figure 1C**).

This review commences by examining the roles fulfilled by macrophages within the microenvironment of HNSCC. Additionally, explain the diverse roles and conditions they play during the development of head and neck tumors. Furthermore, we elaborate on essential therapeutic targets and the most recent related treatment methodologies, with the intent of presenting novel perspectives for forthcoming research and therapeutic strategies targeting macrophages in HNSCC.

## 2 Macrophage involvement in the HNSCC microenvironment

### 2.1 Phagocytosis and secretory regulation

In the tumor microenvironment, macrophages exhibit a dual role in HNSCC. On one hand, they can suppress cancer progression through immune actions, while on the other hand, they play a pro-cancer role by affecting several features of HNSCC, such as immune evasion, promoting invasion and metastasis, participating in angiogenesis, and influencing cancer cell proliferation (27–30).

Phagocytosis is one of the most innate capabilities of macrophages. They can actively engulf abnormal cells within the body, including cancer cells. During this process, macrophages extend pseudopodia to envelop cancer cells in vesicles formed by the cell membrane, creating a structure called a phagosome. Subsequently, this phagosome is internalized within the macrophage (31). This process helps reduce the number of tumor cells, alleviate the tumor burden, and contribute to controlling tumor growth. Currently, in cancer therapy research and development, a new generation of anti-cancer therapies has emerged based on harnessing and enhancing the phagocytic ability of macrophages, such as CAR-engineered cell therapies known as CAR-macrophages (32), which may potentially reshape the landscape of HNSCC treatment in the future.

Macrophages also regulate immune responses and tumor progression by modulating their polarization states through secretory regulation, wherein they secrete cytokines (33). For example, the secretion of certain cytokines may induce macrophages to polarize towards the M1 phenotype, exhibiting characteristics that promote immune responses and anti-tumor effects, such as TNF-α, IL-6, and IL-12 (34). Conversely, other factors may lead to macrophage polarization towards the M2 phenotype, showing features of immune suppression and promoting tumor growth, such as TGF-β and IL-10 (34). Additionally, chemokines such as CXCL9, CXCL10, and CXCL11 can attract T cells and other immune cells, thereby promoting macrophage polarization towards the M1 phenotype (35). The expression levels and activities of these factors are crucial for regulating the polarization status of macrophages, thereby influencing their functions and roles within the tumor microenvironment.

In HNSCC, TAMs typically exhibit an M2-polarized state, promoting immune evasion and growth of the tumor (12). In the early stages of tumors, TAMs release nitric oxide (NO) and reactive oxygen intermediates (ROI), causing DNA damage and genetic instability (36). Afterward, they actively participate in regulating the HNSCC microenvironment through direct secretion. These macrophages can secrete factors that enhance cell migration, including epidermal growth factor, cysteine cathepsins, and matrix metalloproteinases. Through the action of these matrix-degrading enzymes, they facilitate the movement of tumor cells (37). TAMs can also suppress T cell cytotoxicity by secreting IL-10, promote regulatory T cells, leading to immune evasion and tumor proliferation (38). Moreover, they can produce factors that promote the growth of blood vessels within the tumor, such as VEGF-A, VEGF-C, and adrenomedullin, thereby supplying oxygen for tumor development (39).

### 2.2 Exosomes

Extracellular vesicles, also known as exosomes, serve as vesicles originating from tumor cells, immune cells, and various other cell types. They play a role in promoting tumor proliferation, invasion, migration, modulating tumor immunology, fostering angiogenesis, and reprogramming the tumor microenvironment (40). In the progression of head and neck cancer, exosomes serve as a vital means of communication between macrophages and cancer cells. Previous studies have revealed that M1 macrophages secrete exosomes, inhibiting the proliferation, migration, and invasion abilities of head and neck cancer. These exosomes can also induce apoptosis in cancer cells, and HOXA transcripts at the distal tip (HOTTIP), as a tumorigenic specific lncRNA, is a critical molecule in these exosomes, showing the same functionality when overexpressed (41).

Additionally, in oral squamous cell carcinoma, cancer cells secrete exosomes, particularly CMTM6, which induce polarization of M2 macrophages via the ERK1/2 signaling pathway, thereby promoting malignant progression of the tumor. During this process, CMTM6 also enhances the expression of PD-L1, thereby driving tumor migration and invasion (42).

Exosomes extracted from other HNSCC cell lines, including JHU011, SNU1076, and SCC-VII, can significantly induce polarization of M2 macrophages. Exosomes carrying PD-L1 and stimulating HNSCC promote the activation of regulatory T cells (aTregs), further strengthening the positive feedback loop between aTregs and M2 macrophages, ultimately leading to immune escape in tumors (43). The crosstalk mediated by exosomes between macrophages plays a significant role in the complex pathophysiology of head and neck cancer.

### 2.3 Macrophage polarization identification

Traditionally, in HNSCC surgical specimens, TAMs are typically detected using specific antibodies like CD68, CD80, and CD163. CD80+ corresponds to M1 type, while CD163+ corresponds to M2 type (44).

Recently, there has been a shift towards using the mutually exclusive gene expression of CXCL9 and SPP1, along with their ratio, as key features and standards for assessing the polarization level of macrophages within the TME. Bill et al. conducted sequencing and clinical data analysis on 52 patients with head and neck squamous cell carcinoma, revealing that the CXCL9:SPP1 expression ratio, termed CShi or CSlow, is associated with inhibition of certain pro-tumor and anti-tumor effects in head and neck tumors. For instance, CShi tumors are more prone to immune cell infiltration, promoting anti-tumor immunity (45). They propose that evaluating the CXCL9:SPP1 ratio in macrophages could serve as a comprehensive indicator for several critical aspects, such as the presence of anti-tumor immune cells in cancer, gene expression profiles of different tumor-infiltrating cell types, control or progression of communication networks influencing tumors, and the effectiveness of immunotherapy (45). This valuable insight holds profound implications for prospective studies aimed at formulating personalized treatment strategies and prognostic evaluations.

## 3 Diverse macrophage polarization and its implications in the progression of HNSCC

### 3.1 Polarization

Macrophage polarization refers to the distinct activation status of macrophages at a specific moment. This state is determined by their variable expression of surface receptors, secretion patterns, and functional roles. In cancer researches, macrophages typically exhibit an M1 pro-inflammatory profile in the early phases, but they transition to an anti-inflammatory M2 profile in later stages. Macrophage polarization is dynamic, reflecting their adaptability, and it can change in response to a variety of signals from other cells, tissues, and pathogens (46).

In the mice model of oral cancer precursor lesions exposed to nicotine smoke, the degree of M2 polarization at the disease site increased with exposure. Simultaneously, metabolic levels of compounds such as L-nicotine, D-glutamate, arachidic acid, and L-arginine also rose. Some of the mice with heightened M2 polarization even directly developed oral cancer. During this process, there was a decrease in pro-inflammatory factors (iNOS and TNF-α) that induce M1 polarization, resulting in reduced monocyte recruitment to replace them. The polarization shifted towards M2, leading to a significant increase in M2 functional factors like Arg-1 and IL-10. Further, this sustained M2 polarization is indicative of an ongoing immune response, facilitating heightened he activity of Th2 cells and instigating an immunogenic reaction (47).

In head and neck tumors, the Warburg effect manifests as excessive lactate formation, enabling cancer cells to adapt their metabolism to meet the oxygen requirements and the substantial nucleotide, amino acid, and lipid needs for cell proliferation (48). The end product, lactate, is found at higher concentrations in head and neck cancer compared to normal tissue, creating an active environment to promote cancer progression. Notably, the lactate produced by tumor cells has multifaceted effects. It can promote tumor progression by activating pro-inflammatory pathways like IL-23/IL-17 (49), while also inducing monocytes to polarize towards the M2 phenotype, thereby serving as a mediator of immunosuppression to further drive cancer progression (50, 51). Indeed, low pH can reduce the expression of iNOS, CCL2, and IL-6 in M1 macrophages, but increase the expression of M2 macrophage markers in the TME (52). Correspondingly, lactate can promote the M2-like phenotype by activating G-protein-coupled receptor 132 (GPR132) in macrophages, and genetic deletion of Gpr132 in macrophages reduces the M2-like features of tumor-associated macrophages and decreases lung metastasis in a mouse breast cancer model (53). Additionally, lactate can induce the expression of the enzyme ARG1 which indicates lactate can transform macrophages into immunosuppressive macrophages and promote M2-macrophage polarization in mice (54). The intratumoral lactate levels in human HNSCC are associated with the polarization of M2-like macrophages in the TME as well. When lactate levels in the tumor are low, more macrophages accumulate at the tumor site. Conversely, under conditions of high lactate concentration, monocyte migration is inhibited, preventing effective macrophage infiltration into lactate-rich tumors, but it promotes their polarization towards the M2 phenotype. However, unlike in mice models, both lactate and M2-polarization levels are not associated with the expression of ARG1 in human macrophages (55).

Moreover, in the interaction between cancer cells and macrophages in HNSCC, cancer cells release Apelin peptide, which promotes the polarization of M2 macrophages. Inhibiting the release of Apelin peptide by cancer cells leads to an increase in pro-inflammatory responses in co-cultured macrophages, resulting in a significant upregulation of genes like IL-1β, IL-6, and TNF-α, along with a marked reduction in anti-inflammatory cytokine levels. In the Apelin (+) group, pro-inflammatory factors are decreased, while anti-inflammatory factors are heightened (56).Tumor-derived extracellular vesicles expressing TGF-β also play a significant role in the crosstalk between tumor cells and macrophages in the HNSCC TME. These extracellular vesicles induce polarization and chemotaxis of human macrophages and also reprogram the function of primary human macrophages. This reprogramming results in increased secretion of pro-angiogenic factors, including Angiopoietin-2, MMP-9, PD-ECGF, and TIMP-1, and a shift toward a pro-angiogenic phenotype. Upon injection into mice with oral cancer induced by 4-nitroquinoline 1-oxide (4-NQO), these vesicles promote tumor angiogenesis, enhance infiltration of M2-like macrophages, and accelerate tumor progression (28).

The mechanisms through which M2-polarized cells regulate the progression of head and neck cancer are intricate. TAMs enhance the stemness of head and neck cancer cells by activating the PI3K-4EBP1 pathway. Additionally, TAMs interact with head and neck cancer cells through the CD44-VCAM-1 pathway, ultimately boosting the invasive capabilities of cancer cells (57). Furthermore, it has been demonstrated that M2 polarization can increase the expression of PD-L2 in TAMs, leading to immune evasion and tumor progression through the PD-1 signaling pathway (58).

### 3.2 Re-polarization

In general, M1 macrophages provide immune protection by releasing pro-inflammatory cytokines, whereas M2 macrophages exhibit anti-inflammatory properties that aid in tissue remodeling and tumor advancement (59). In cancer research, the differentiation of macrophages into M1 type from the alternative M2 type, a process known as macrophage repolarization, is a promising approach in contemporary cancer immunotherapy. Repolarizing TAMs from M2-to-M1 is considered a prospective therapeutic strategy.

To reprogram TAMs without altering the M1/M2 polarization balance within healthy organs, Xiao et al. developed a micelle nano-therapy. They released M2-targeted antagonists after exposure to the acidic tumor microenvironment, co-delivering inhibitors like STAT6 to effectively achieve M2-to-M1 repolarization, thereby inhibiting tumor growth and metastasis (60). Additionally, Wu et al. have coupled targeted drugs with tumor-specific STING agonists, finding that within the tumor microenvironment, M2 repolarizes towards M1 (61). Furthermore, statins have been found to inhibit proliferation of recurrent/metastatic HNSCC cells, enhance T cell cytotoxicity against tumor cells, and promote M2-to-M1 macrophage repolarization (62). Statins, known for their tolerability and affordability, may further enhance responses to PD-L1 checkpoint blockade and other HNSCC immunotherapies, although this potential remains to be fully explored.

Simultaneously, macrophage repolarization often broadly refers to macrophages polarizing towards different functional directions. In the previously mentioned exosomes, M1 exosomes and HOTTIP induced M1 repolarization within the tumor microenvironment, encompassing macrophage repolarization.

## 4 Main targets for macrophage targeting in HNSCC

### 4.1 STAT3

Signal transducer and activator of transcription 3 (STAT3) is frequently overactivated in various human cancers, serving as a crucial signaling node in tumor cells and the cellular components of the TME, especially in tumor-infiltrating immune cells (63). Radiation therapy, a commonly used treatment for HNSCC, aims to utilize high-energy radiation to selectively kill or control the growth of cancer cells, reducing tumor size or eliminating the tumor altogether (64). It is often employed as a treatment option for patients who are not suitable candidates for surgery.

In the circulatory system of HNSCC patients undergoing radiotherapy, there is an accumulation of therapy-resistant bone marrow cells, which affects the efficacy of radiation therapy (65). Moreira et al. found that targeting STAT3 in TAMs can enhance the therapeutic effects of radiation therapy for HNSCC. They employed the CpG-STAT3ASO strategy to target STAT3 in HNSCC-related macrophages in conjunction with TLR9 triggering. This approach can overcome radiation resistance in tumors of both HPV-positive and HPV-negative mice. The combined treatment results in reduced residual M2 macrophages in the tumor and the recruitment of activated M1 macrophages to the tumor-draining lymph nodes (TDLNs).

A single-cell transcriptomic study of oral squamous cell carcinoma has revealed an enrichment of the IL-6/JAK2/STAT3 axis in the tumor microenvironment, particularly in cell populations like macrophages, in samples induced by chemotherapy and other treatments (66). Additionally, the phosphorylation level of STAT3 can modulate the response of regulatory T cells (Tregs) to radiation therapy in head and neck cancer (67). These findings indicate that STAT3 could serve as a significant combinatorial therapeutic target to enhance the efficacy of radiotherapy and chemotherapy in head and neck cancer.

Targeting STAT3 in current research offers several advantages, including improving immune dysregulation in the tumor microenvironment, reducing endogenous proliferation of tumor cells, and enhancing the anti-tumor effects of tumor-infiltrating immune cells, among others (68, 69). As a potential target for cancer treatment, the current drug development efforts against STAT3 involve direct inhibition using peptides, small molecules, and decoy oligonucleotides (70–73), or indirect inhibition through blocking upstream signaling pathways such as the IL-6 and JAK2 pathways (74, 75).

### 4.2 CCL2/CCR2

CC-chemokine receptor 2 (CCR2) is primarily expressed in monocytes and macrophages and has a strong pro-inflammatory function (76). This has led to the development of CCR2 antagonists aimed at inhibiting unnecessary immune responses in inflammation and autoimmune diseases. Paradoxically, in the tumor microenvironment, CCR2-expressing monocytes and macrophages can strongly suppress immune responses (77). In recent years, researchers have explored strategies using CCR2 antagonists to selectively attract suppressive monocytes and macrophages into the tumor, with the goal of altering the tumor microenvironment and enhancing the immune system’s ability to combat cancer (78).

While the mice model of HNSCC treated with radiotherapy, there showed an increase in the production of the chemotactic factor CCL2 in tumor cells, leading to the accumulation of CCR2-dependent TNF-α-producing monocytes/macrophages and CCR2+ Tregs (79). CCL2/CCR2 could potentially serve as clinical candidates for radioimmunotherapy to counteract the radio-protective effects of macrophages and Treg cells. Currently, synthetic inhibitors of CCL2, Bindarit(Bnd) (80) and Carlumab(CNTO 888) (81), as well as CCR2 antagonists RS-50439 and MLN1202, have been developed for targeted disruption of CCL2/CCR2 signaling to intervene in the progression of various tumor types (82–85).

### 4.3 NRF2

NRF2, encoded by the NFE2L2 gene, plays a crucial role in maintaining cellular redox homeostasis, regulating immune responses, and detoxifying drugs (86, 87). Activation of NRF2 can lead to metabolic reprogramming, enhancing tumor proliferation, suppressing various forms of stress, and promoting immune evasion (88).

NRF2 is upregulated in HNSCC, and its expression levels are positively correlated with malignancy (89, 90). Carcinogens such as nicotine and arecoline can trigger c-myc-driven NRF2 activation in HNSCC cells, reprogramming the pentose phosphate pathway metabolism in the tumor microenvironment (90). In this metabolic pathway, glucose-6-phosphate dehydrogenase (G6PD) and transketolase (TKT) are key downstream effectors driven by NRF2, contributing to the progression of head and neck squamous cell carcinoma.

Mutations in the NRF2-encoding gene NFE2L2 can result in radiation resistance in HNSCC. Notably, in HNSCC patients undergoing radio chemotherapy, NFE2L2 mutations are significantly linked to a heightened risk of local treatment failure. In immunocompetent mice, tumors carrying NFE2L2 mutations displayed increased resistance to radiation compared to tumors with the wild-type NRF2. However, this discrepancy was less pronounced in immunodeficient mice. NFE2L2 enhances radiation resistance by diminishing the presence of M1-polarized macrophages (91).

Previously, researchers have attempted to inhibit NRF2 by studying the natural inhibitory protein Kelch-like ECH-associated protein 1 (KEAP1) that targets NRF2 (92, 93). Additionally, in order to discover new NRF2 inhibitors for targeted therapy, Singh et al. conducted a quantitative high-throughput screening in the small molecule library MLSMR and identified ML385 as a probe molecule that binds to NRF2 and inhibits its downstream target gene expression (94).

### 4.4 CD47

CD47 is a widely expressed cell surface protein that acts as a ligand for signal regulatory protein alpha (SIRPα) on macrophages, which, in turn, inhibits phagocytosis (95). Previous studies in various preclinical models have demonstrated that blocking the CD47-SIRPα pathway can enhance phagocytic functions, demonstrating significant anti-tumor efficacy across multiple tumor types (96, 97).

Regarding a macrophage-mediated anti-tumor immunotherapy strategy based on gene-edited nanoparticles: the first step involves blocking the CD47-SIRPα pathway, and the second step is to repolarize tumor-associated macrophages (98). Additionally, Ni and colleagues discovered in preclinical models using the IBI188 drug to block the CD47-SIRPα pathway that angiogenesis can, to some extent, limit the effectiveness of anti-CD47 antibodies against tumors. Combining anti-angiogenesis therapies with CD47 blockade can achieve higher therapeutic efficacy (99).

Recently, Lee et al. conducted macrophage phagocytosis experiments on the HN31R head and neck cancer cell line and found that the downregulation of Tristetraprolin (TTP) can induce sustained overexpression of CD47, which, in turn, inhibits the phagocytosis of head and neck cancer cells (100). Furthermore, when CD47 was expressed *in vitro* in HNSCC cell lines, both M1 and M2 macrophages exhibited a certain degree of phagocytic potential (101). However, under conditions where CD47 was inhibited, the phagocytic ability of M1 enhanced, while M2 did not (101). In summary, CD47-positive oral squamous cell carcinoma cells primarily inhibit M1 phagocytosis, leading to immune evasion.

Currently, several antibodies targeting CD47 have entered clinical trials, such as Hu5F9-G4, TTI-621, and others, for the treatment of both solid tumors and hematologic malignancies (102, 103).

### 4.5 TGF-β

Transforming growth factor-β (TGF-β) is a widely recognized immunosuppressive factor, playing a role in restraining excessive inflammatory responses (104–106). Additionally, TGF-β triggers macrophage M2 polarization, contributing to the alleviation of inflammation mediated by macrophages (105, 107).

PD-1 blockade therapy in the treatment of HNSCC has demonstrated a significant extension of survival in recurrent/metastatic (R/M) patients, coupled with favorable safety profiles (108, 109). However, numerous challenges persist, with a substantial portion of cancer patients exhibiting suboptimal responses to PD-1 monotherapy (110). The crucial role of TGF-β in the delicate balance between immunity and tolerance among non-responsive patients to PD-1 monotherapy has been identified (111, 112). TGF-β modulates the cancer immune cycle by altering T cell proliferation, activation, differentiation, and impeding the activity of dendritic cells and natural killer cells (113). Combining anti-TGF-β with anti-PD-1 therapy has proven effective in overcoming resistance in immune rejection models (114, 115). Subsequently, Yi et al. developed the bispecific antibody (BsAb) YM101 which targeting both TGF-β and PD-L1 (116). They observed potent anti-tumor activity of this drug in immune-inflammatory and immunosuppressive models of diverse tumors (117). Additionally, a TGF-β/PD-L1 specific antibody, the drug BiTP, has been developed and demonstrated promising anti-tumor efficacy in both *in vitro* and *in vivo* experiments (118). Simultaneously, Matos et al. have recently engineered a Polyoxazoline-Based nano-vaccine carrying a TGF-β expression regulator in combination with a PD-1 inhibitor. This combination exhibits synergistic anti-tumor effects and holds significant potential in improving the immunotherapeutic outcomes for solid cancer patients (119).

### 4.6 Other targets

The regulation of macrophage proliferation, differentiation, and survival hinges on the control of CSF1R and its ligands. A multitude of preclinical investigations have underscored that the inhibition of CSF1R leads to a decreased density of TAMs, resulting in the inhibition of tumor growth and heightened sensitivity to chemotherapy (120, 121). Besides, elevated expression of CSF1R leads to increased lactate levels in HNSCC, reduces the presence of tumor-infiltrating macrophages, and promotes the induction of M2-like macrophage polarization within the tumor (55). The drugs targeting CSF1/CSF1R, such as PLX3397 and HMPL-012, have been proven to be effective in other tumors, including tenosynovial giant cell tumor and neuroendocrine tumors (122, 123).

Moreover, high expression of RACK1 in oral squamous cell carcinoma (OSCC) is associated with increased infiltration of M2 macrophages (124). OSCC cells that overexpress RACK1 promote M2-like macrophage polarization through the regulation of NF-kappa B, leading to an increase in the proportion of M2-like macrophages in xenograft mouse models (27). The corresponding targeted drug is M435-1279, a critical ubiquitin-conjugating enzyme E2T (UBE2T) inhibitor that catalyzes the proteasomal degradation of RACK1, which also has certain prospects for future applications (125). Those typical targets, pathways, and associated drugs of macrophages in the progression HNSCC are shown in **Table 1**.

**Table 1:** Typical targets, pathways, and associated drugs of macrophages in HNSCC progression.

Targets	Pathways	Drugs	References
STAT3	IL-6/JAK2/STAT3	LL1	(70)
		SD-36	(72)
		W1131	(73)
CCR2	CCL2/CCR2	Bnd	(80)
		CNTO 888	(81)
		RS-50439	(83)
		MLN1202	(84)
NRF2	KEAP1/NRF2	ML385	(94)
CD47	CD47/SIRPα	IBI188	(99)
		Hu5F9-G4	(102)
		TTI-621	(103)
TGF-β	TGF-β/PD-L1	YM101	(117)
		BiTP	(118)
CSF1R	CSF1/CSF1R	PLX3397	(122)
		HMPL-012	(123)
RACK1	RACK1/NF-kappa B	M435-1279	(125)

## 5 Applications of macrophages in the treatment of HNSCC

### 5.1 Conventional immunotherapy targeting

Macrophages are an important target in current checkpoint blockade immunotherapy, suppressing adaptive immune responses by expressing inhibitory counter-receptors such as PD-L1 and PD-L2. Certain chemotherapy drugs, like anthracyclines, induce the release of tumor antigens and co-stimulatory molecules, a process referred to as immunogenic cell death, engaging macrophages in a productive cancer immune cycle (126). There are also other cell-depleting therapies aimed at targeting macrophages (127). Specific strategies focused on macrophages have partly entered clinical assessment, including monocyte-derived macrophages used for cellular therapy, either through targeted recruitment and differentiation or functional reprogramming via activation or inhibition of checkpoint receptors.

In the treatment of recurrent/metastatic HNSCC patients, checkpoint inhibitors have demonstrated their effectiveness (128). However, the majority of patients do not benefit from these drugs (129). To enhance the efficacy of checkpoint inhibitors, Sato-Kaneko F et al. have established HNSCC models and employed a combination of TLR agonists and PD-1 blockade (130). They found that this approach could activate TAMs, induce tumor-specific adaptive immunity, and thus inhibit primary tumor growth and prevent metastasis. Notably, treatment with TLR7 agonists increased the M1/M2 ratio and promoted the generation of tumor-specific immune factors.

To enhance the immunotherapeutic efficacy in HNSCC, Wu et al. developed an injectable nano-composite hydrogel (131). This hydrogel is created by incorporating imiquimod-encapsulated CaCO3 nanoparticles (RC) and a cancer cell membrane (CCM)-coated mesoporous silica nanoparticle within a polymer framework (PLGA-PEG-PLGA). These components include a peptide-based protein hydrolysis targeting chimera (PROTAC) against BMI1 paclitaxel (PepM@PacC). The injectable hydrogel can selectively manipulate tumor-associated macrophages, further activating T cell immune responses.

### 5.2 Engineering macrophages

In response to the phagocytic and pro-inflammatory actions of M1 macrophages on tumor cells, engineered macrophages targeting cancer cells as carriers for anti-tumor therapy have been developed to modulate the tumor microenvironment (132).

For example, controlled-release biomimetic or macrophage membrane-coated nanoparticles have been developed for cancer therapy to respond to the TME (21). Rao et al. engineered cell membrane-coated magnetic nanoparticles (gCM-MNs) to enhance the affinity between the genetically overexpressed SIRPα variant on gCM shells and CD47, effectively blocking the CD47-SIRPα pathway and preserving macrophage’s ability to phagocytose cancer cells (133). Meanwhile, these magnetic nanoparticles promote M2-to-M1 repolarization in the tumor microenvironment of B16F10 melanoma mice model and the triple negative breast cancer 4T1 mice model, blocking the process of tumor cells secreting colony-stimulating factors to polarize tumor-associated macrophages into tumor-promoting M2 macrophages. This synergistically enhances macrophage phagocytosis of cancer cells and triggers anti-tumor T-cell immunity. This method effectively activates macrophages for anti-tumor immunotherapy. In addition, macrophage membrane-coated nanoparticles (cskc-PPiP/PTX@Ma) developed by Zhang et al. show enhanced therapeutic effects, homing to tumor sites and gradually controlling drug release in response to the acidic pH changes in the tumor microenvironment, releasing the hydrophobic anti-cancer drug paclitaxel to kill cancer cells. Testing the administration capability and therapeutic effects of this formulation in an orthotopic breast cancer-bearing mice model, this combination of a biomimetic cell membrane and a cascade-responsive polymeric nanoparticle yielded significant results (134).

Furthermore, Rayamajhi and colleagues developed hybrid exosomes (HE) with a size smaller than 200nm by hybridizing exosomes extracted from mouse macrophages with synthetic liposomes (135). They loaded a water-soluble doxorubicin into these HE, increasing the toxicity of drug-loaded HE to cancer cells and enabling drug release in the acidic tumor microenvironment. These macrophage-derived mimetic exosome vesicles effectively deliver bioactive molecules to recipient cells, making them suitable for drug delivery and therapy in cancer.

### 5.3 CAR-macrophage

Chimeric antigen receptor (CAR)-T cell therapy is an early cell-based immunotherapy designed to prevent tumor cells from evading recognition by T cell receptors. This method has been successfully used to treat hematologic malignancies, but its effectiveness in solid tumors remains limited (136). In the tumor microenvironment, macrophages, as the most abundant innate immune cells, can infiltrate solid tumor tissues and interact with almost all other cell types (137). Therefore, researchers are attempting to use CAR-modified macrophages (CAR-M) to combat solid tumors.

The first-generation CAR-M cells primarily utilize the characteristics of macrophages, focusing on their phagocytic function (138, 139). In contrast, second-generation CAR-M cells, in addition to retaining the features of the first generation, also aim to improve the presentation of tumor-associated antigens and T cell activation. In this scenario, Klichinsky et al. design murine or human macrophages through chimeric vectors and then obtain the drug after *in vitro* expansion, concentration, and purification (140). Currently, third-generation CAR-M cells are being designed by reprogramming CAR-M cells *in vivo* using non-viral vectors (141). There has been an approach to fuse nanobiotechnology with CAR-M cells, using nanocarriers to deliver the encoded CAR and interferon-gamma genes to macrophages *in vivo*, with the aim of further enhancing anti-tumor efficacy by repolarizing M2-polarized macrophages into M1 macrophages (142).

The progress of CAR-M therapy in HNSCC is currently quite limited. However, with the continuous iteration of CAR-M technology and the advancement of macrophages in head and neck squamous cell carcinoma, this field holds tremendous potential for application.

## 6 Discussion and prospects

Macrophages exhibit a high degree of plasticity in response to various microenvironments within normal human tissues, inflammatory stimuli, and tumor tissues. This functional diversity results in various characteristics within macrophages, making their categorization challenging. Currently, macrophages are broadly categorized into two phenotypes, M1 and M2, which are associated with pro-inflammatory and anti-inflammatory properties, respectively. Tumor-associated macrophages represent the complex interplay of various cell types within the TME and can exhibit M1 or M2 characteristics under the influence of different TME stimuli. Typically, M1-like TAMs that promote an inflammatory response against tumor cells often exhibit anti-cancer effects, while M2-like TAMs tend to support tumorigenesis (143).

**Figure 2:**
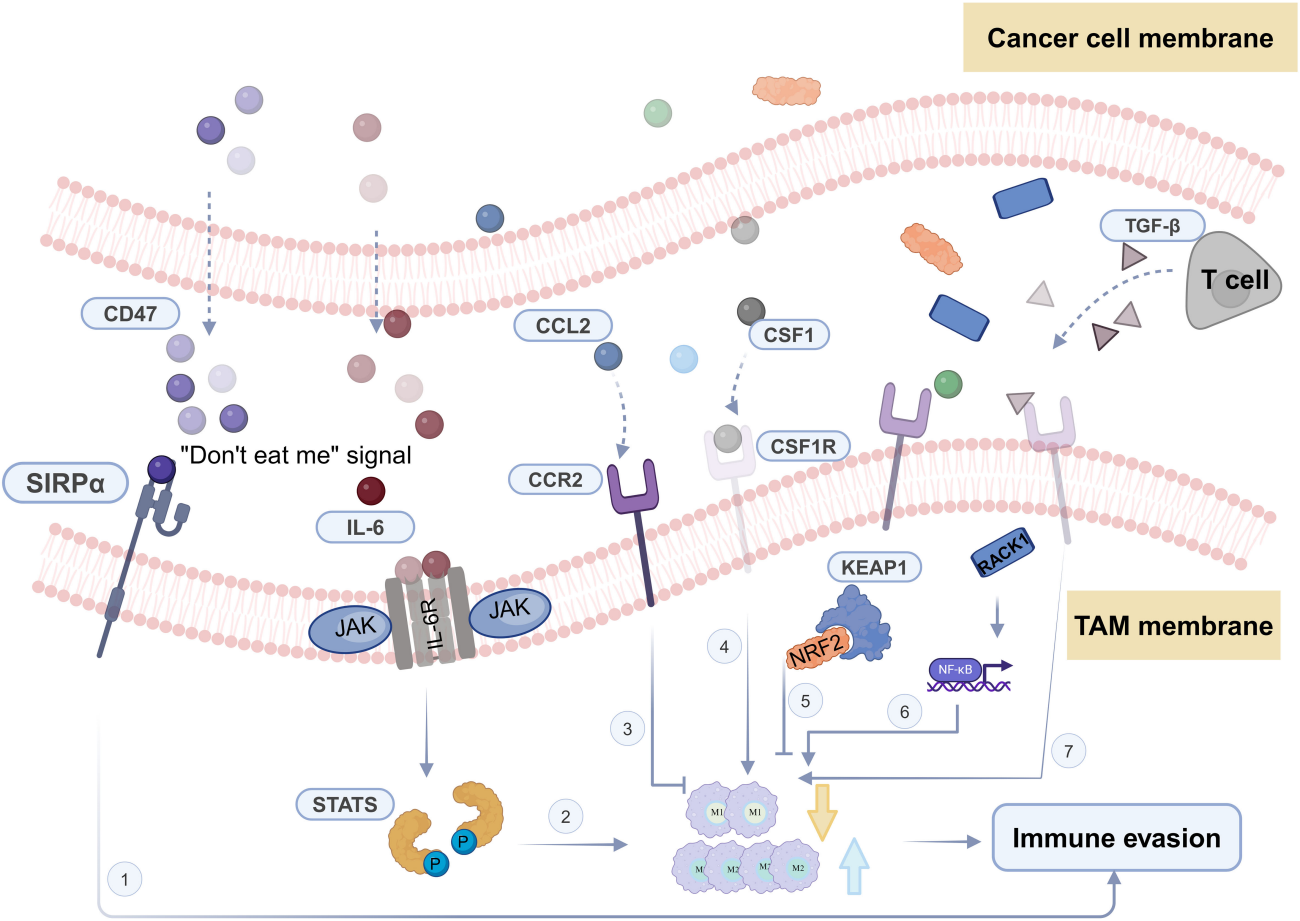
Crucial pathways and key molecules of tumor-associated macrophages in TME immune evasion.

Head and neck squamous cell carcinoma, being an invasive malignant tumor, is characterized by high incidence and low survival rates. Treatment options for HNSCC are limited, typically involving local surgery, radiation, and chemotherapy (144). The development of immunotherapy has harnessed the collaborative role of the TME in HNSCC progression. By understanding the processes of tumor cell evolution and immune evasion (**Figure 2**), immunotherapy demonstrates effective anti-cancer properties through the manipulation of self-immunogenicity or the expression of immune inhibitory mediators, ultimately enhancing the survival rates of HNSCC patients. However, it’s worth noting that fewer than 20% of patients exhibit sustained responses to these treatments (145).

Within the HNSCC microenvironment, TAMs, being the most abundant group of innate immune cells, play a role in mediating immunosuppressive effects on adaptive immune cells in the TME. The polarization state of TAMs can be influenced by various signals like nicotine, Apelin peptide, and lactate. This polarization state has a strong connection with the development and immune evasion in head and neck cancer, although it doesn’t necessarily impede immune responses. In the context of head and neck cancer, the M2 polarization of macrophages can impact tumor stemness, invasiveness, and the mechanisms of immune evasion. Consequently, inhibiting M2 polarization and promoting M2-to-M1 repolarization have emerged as crucial strategies that leverage the remarkable plasticity of macrophages in anti-cancer efforts. Building upon this foundational theory, more effective immunotherapeutic approaches have been further explored, including the engineering of macrophages and the utilization of CAR-M technology to eliminate HNSCC cells.

Targeting TAMs and HNSCC remains an ongoing and challenging endeavor in progress. Macrophages play a crucial dual role in different anticancer modalities, as they are actively involved not only in chemotherapy, radiation therapy, and immune checkpoint blockade (ICB) immunotherapy, as mentioned above, but also in anti-angiogenesis and hormone therapy. For instance, in one study, it was found that metformin reduces the accumulation of M2-TAMs in the tumor microenvironment, impeding M2-like macrophage-induced angiogenesis promotion. On the other hand, melatonin indirectly inhibits tumor angiogenesis by increasing the accumulation of M1-TAMs (146). Consequently, developing more precise targeted treatment strategies and exploring the potential of macrophage-based therapies are all research directions for further improving HNSCC survival rates and refining the approaches to HNSCC treatment in the future.

This review presents a comprehensive overview of the immune-regulatory roles played by macrophages in HNSCC. It delves into the diverse polarization states of macrophages within the tumor microenvironment and explores potential therapeutic strategies for repolarization. Recent years have witnessed significant progress in research targeting critical macrophage-related factors, along with substantial advancements and refinements in macrophage-based therapies for head and neck cancer. These developments aim to boost the efficacy of immunotherapy for HNSCC. Through this contribution, our objective is to advance macrophage-related therapeutic strategies for HNSCC, revealing more effective potential treatment methods in this evolving era.

## Author contributions

CL: Writing – review & editing, Writing – original draft. YC: Writing – review & editing, Visualization, Validation, Investigation. YZ: Writing – review & editing, Visualization, Validation. SG: Writing – review & editing, Supervision, Funding acquisition. YH: Writing – review & editing, Methodology. JH: Writing – review & editing, Methodology. ZS: Writing – review & editing, Supervision, Project administration, Funding acquisition.
